# Gynæcology

**Published:** 1878-09

**Authors:** 


### GYNÆCOLOGY.

DIAGNOSIS OF MALIGNANT OVARIAN TUMORS AND MALIGNANT PERITONITIS.—Foulis.—(*Br. Med. Jour.*, July 20, 1878.)

In one of his lectures on the above subject, Prof. Foulis, of Edinburgh, sums up as follows:

Malignant ovarian tumors are generally surrounded by ascitic fluid; this should always be carefully examined with the microscope. If in it we find a large number of sprouting, vigorously-growing cells, some of which masses may be seen by the naked eye, we may safely conclude that the peritoneum is the seat of a most serious disease, and that there are numerous adhesions between the tumor and neighboring parts, which will prevent its complete removal by ovariotomy. If these masses be found in bloody ascitic fluid, we may conclude that there are vascular, villous growths on the peritoneum; and if, in addition, the glands in the groin or abdominal parietes are enlarged, the tumor and peritoneal growths are certainly carcinomatous.

As an additional means of diagnosis the tumor itself may be punctured, under antiseptic precautions, with an aspirator needle, and a minute portion or some of its contents be withdrawn. The finding of sprouting masses of epithelium in ovarian cysts is not of much value, as such tumors may be often removed; but if they be found in the cysts of a tumor and in the ascitic fluid also, it is almost certain that serious adhesions exist between the tumor and adjacent parts, and that there are nodular growths on the peritoneum.

PATHOLOGY OF MEMBRANOUS DYSMENORRHŒA.—Cory.—(*Obstetrical Journal*, June, 1878.)

Dr. Cory related to the London Obstetrical Society the case of a patient who menstruated normally at fifteen, but never passed any membrane till after her marriage, at the age of thirty. Within two years thereafter, she had three miscarriages early in pregnancy. She almost invariably passed membranes with each subsequent period, except when she happened to have been apart from her husband. One interval of nine months without marital approach was passed without the distressing features detailed above.

He thought the case favored the view that *her* menstrual epochs were due to the abortion of an impregnated ovum, together with its nidus, mucous membrane of the uterus—the decidua.

In discussion of the case following its report, the opinions were expressed that the term membranous dysmenorrhœa was inappropriate for such a case; that the ovum belonged to the period previous to the one during which it was expelled, otherwise no decidua could have formed; that ovulation was at best an irregular function, and that hypernidation might be produced by the uterine hyperæmia set up by marital life; that spermatozoa may live for some days, and impregnation take place after a menstrual period though connection has occurred before it.

